# Comment on “The role of apparent diffusion coefficient values in diagnosing prostate cancer for patients with equivocal PI-RADS 3 lesions: a multicenter retrospective study”

**DOI:** 10.1097/JS9.0000000000004778

**Published:** 2026-01-20

**Authors:** Anguo Wang

**Affiliations:** Reproductive Medicine Center, Hainan Women and Children’s Medical Center, Haikou, China

*To the Editor,*

Wang *et al* address a common and clinically consequential dilemma: PI-RADS 3 lesions are frequent, yet the yield of clinically significant prostate cancer (csPCa) is modest, exposing many men to potentially avoidable biopsy morbidity[1]. This is especially relevant as MRI-first diagnostic pathways become increasingly embedded in contemporary practice^[2,3]^. This commentary was prepared in line with the TITAN 2025 guideline to promote transparent and accountable reporting[4].

In a multicenter cohort of 460 men with PI-RADS 3 lesions on biparametric MRI, only 13.5% harbored csPCa, underscoring the need for reliable “biopsy-or-defer” refinement within the PI-RADS v2.1 framework[1]. The authors’ principal contribution is the demonstration that ADCmin, measured via a standardized ROI protocol, outperforms other ADC descriptors for csPCa discrimination. On multivariable analysis, prostate volume, ADCmin, and peripheral-zone location emerged as independent predictors, and the combined model achieved strong discrimination (AUC 0.893 internally; external AUC 0.800–0.933)[1].

The most actionable element is the proposed rule-out strategy. Using prostate volume <50 mL or ADCmin <0.65 × 10^−3^ mm^2^/s to trigger biopsy delivered very high sensitivity (93.5%) and NPV (98.3%) for csPCa, while a three-tier risk approach could reduce biopsies by ~51% at the cost of missing 0.9% csPCa cases[1]. In the context of MRI-directed diagnosis – where randomized evidence shows MRI triage can avoid biopsy in men with non-suspicious MRI while improving detection of csPCa – such calibrated deferral rules for the equivocal PI-RADS 3 category are clinically attractive^[2,3]^.

Several caveats deserve emphasis before broad implementation. ADC values are sensitive to scanner vendor, sequence parameters, and post-processing; therefore, portability of a single ADCmin threshold across sites remains uncertain without harmonization. PI-RADS v2.1 aims to standardize acquisition and interpretation, but inter-reader reproducibility of ROI placement and ADCmin extraction should be quantified formally, as this will determine real-world reliability^[1,5]^. Finally, biopsy-based endpoints and retrospective design limit inference; prospective validation with predefined thresholds and clinically integrated decision rules (e.g., alongside PSA density and lesion volume) is needed to ensure safe biopsy deferral.

Overall, Wang *et al* provide strong multicenter evidence that ADCmin, combined with prostate volume and zonal anatomy, can meaningfully refine PI-RADS 3 decision-making and potentially reduce unnecessary biopsies[1].

## Data Availability

No data were used in this Letter to the Editor.
